# Supplementary material for: Outcomes of Transcatheter Aortic Valve Implantation Comparing Medtronic’s Evolut PRO and Evolut R: A Systematic Review and Meta-Analysis of Observational Studies
Source: Int J Environ Res Public Health. 2023 Feb 15;20(4):3439. doi: 10.3390/ijerph20043439 (PMC9967567; doi:10.3390/ijerph20043439)

# Outcomes of transcatheter aortic valve implantation with Medtronic's Evolut PRO compared to Evolut R. Systematic review and meta-analysis of observational studies.

## SUPPLEMENTARY MATERIAL

| **Supplementary Table S1**. MOOSE Checklist for Meta-analyses of Observational Studies | | |
|---|---|---|
| Item No | Recommendation | Reported on Page No |
| | Reporting of background should include | |
| 1 | Problem definition | 1 |
| 2 | Hypothesis statement | NA |
| 3 | Description of study outcome(s) | 1 |
| 4 | Type of exposure or intervention used | 1 |
| 5 | Type of study designs used | 1 |
| 6 | Study population | 1 |
| | Reporting of search strategy should include | |
| 7 | Qualifications of searchers (eg, librarians and investigators) | Title page |
| 8 | Search strategy, including time period included in the synthesis and key words | 4, Figure 1 |
| 9 | Effort to include all available studies, including contact with authors | 2 |
| 10 | Databases and registries searched | 2 |
| 11 | Search software used, name and version, including special features used (eg, explosion) | NA |
| 12 | Use of hand searching (eg, reference lists of obtained articles) | NA |
| 13 | List of citations located and those excluded, including justification | NA |
| 14 | Method of addressing articles published in languages other than English | NA |
| 15 | Method of handling abstracts and unpublished studies | NA |
| 16 | Description of any contact with authors | NA |
| | Reporting of methods should include | |
| 17 | Description of relevance or appropriateness of studies assembled for assessing the hypothesis to be tested | NA |
| 18 | Rationale for the selection and coding of data (eg, sound clinical principles or convenience) | NA |
| 19 | Documentation of how data were classified and coded (eg, multiple raters, blinding and interrater reliability) | NA |
| 20 | Assessment of confounding (eg, comparability of cases and controls in studies where appropriate) | NA |
| 21 | Assessment of study quality, including blinding of quality assessors, stratification or regression on possible predictors of study results | Supplementa ry Table 2 |
| 22 | Assessment of heterogeneity | 3 |
| 23 | Description of statistical methods (eg, complete description of fixed or random effects models, justification of whether the chosen models account for predictors of study results, dose-response models, or cumulative meta-analysis) in sufficient detail to be replicated | 3 |
| 24 | Provision of appropriate tables and graphics | yes |
| | Reporting of results should include | |
| 25 | Graphic summarizing individual study estimates and overall estimate | NA |
| 26 | Table giving descriptive information for each study included | Table 2 |

| | | |
|---|---|---|
| 27 | Results of sensitivity testing (eg, subgroup analysis) | Supplementary Figure 4 |
| 28 | Indication of statistical uncertainty of findings | NA |
| **Item No** | **Recommendation** | **Reported on Page No** |
| | Reporting of discussion should include | |
| 29 | Quantitative assessment of bias (eg, publication bias) | NA |
| 30 | Justification for exclusion (eg, exclusion of non-English language citations) | Figure 1 |
| 31 | Assessment of quality of included studies | Supplementary Table 2 |
| | Reporting of conclusions should include | |
| 32 | Consideration of alternative explanations for observed results | 10 |
| 33 | Generalization of the conclusions (ie, appropriate for the data presented and within the domain of the literature review) | 11 |
| 34 | Guidelines for future research | NA |
| 35 | Disclosure of funding source | 11 |

*From*: Stroup DF, Berlin JA, Morton SC, et al for the Meta-analysis Of Observational Studies in Epidemiology (MOOSE) Group. Meta-analysis of Observational Studies in Epidemiology. A Proposal for Reporting. JAMA 2000;283:2008-2012.

**Supplementary Table S2**. Bias assessment

| Study (matched) | Bias due to confounding | Bias in selection of participants into the study | Bias in measurement of interventions | Bias due to departures from intended interventions | Bias due to missing data | Bias in measurement of outcomes | Bias in selection of reported result | Overall bias |
|---|---|---|---|---|---|---|---|---|
| Forrest JK et al. 2020 [21] | Serious | Critical | Low | Low | Moderate | Serious | Low | MODERATE- |
| Hellhammer K et al. 2018 [22] | Serious | Critical | Low | Low | Low | Moderate | Low | MODERATE+ |
| Loewenstein I et al. 2022 [25] | Serious | Critical | Low | Low | Low | Serious | Low | MODERATE |

| Study (unmatched) | Bias due to confounding | Bias in selection of participants into the study | Bias in measurement of interventions | Bias due to departures from intended interventions | Bias due to missing data | Bias in measurement of outcomes | Bias in selection of reported result | Overall bias |
|---|---|---|---|---|---|---|---|---|
| Alvarado T et al. 2021 [19] | Serious | Cirtical | Low | Low | Low | Low | Low | MODERATE++ |
| Dallan LAP et al. 2021 [20] | Serious | Cirtical | Low | Low | Low | Serious | Low | MODERATE |
| Kalogeras K et al. 2020 [23] | Serious | Critical | Low | Low | Low | Serious | Moderate | MODERATE- |

| | | | | | | | | |
|---|---|---|---|---|---|---|---|---|
| Kroon HG et al. 2021 [24] | Serious | Cirtical | Low | Low | Low | Moderate | Moderate | MODERATE |
| Modolo R et al. 2020 [26] | Serious | Critical | Low | Low | Low | Low | Low | MODERATE++ |
| Rao G et al. 2019 [27] | Serious | Critical | Low | Low | Serious | Moderate | Low | MODERATE- |
| Regazzoli D et al. 2019 [28] | Serious | Critical | Low | Low | Low | Serious | Moderate | MODERATE- |
| Schmidt S et al. 2022 [29] | Serious | Critical | Low | Low | Low | Serious | Low | MODERATE |

| Supplementary Table S3. Inclusion (choice of procedure type), exclusion criteria and choice of valve type. | | | |
|---|---|---|---|
| Study [ref] | Inclusion criteria/ Selection criteria for the procedure | Exclusion criteria | Selection criteria for the valve |
| Alvarado T et al. 2021 [19] | Patients presenting symptomatic (NYHA class >2) severe aortic stenosis (defined as aortic valve area (AVA) <1 $cm^2$ or indexed AVA <0.6 $cm^2/m^2$ who were deemed intermediate or high risk (STS >3) for SAVR. | Severe mitral regurgitation and presence of an aortic annulus size outside the limits recommended for the implant (perimeter derived diameter <18 mm or >26 mm). | Not reported. |
| Dallan LAP et al. 2021 [20] | Data from patients who underwent a TAV-in-SAV procedure using an Evolut R or Evolut PRO valve between April 2015 and June 2019 retrieved from the TVT Registry. | Not reported. | Not reported. |
| Forrest JK et al. 2020 [21] | TVT Registry database for patients with native tricuspid aortic valve stenosis treated with Medtronic self-expanding transcatheter aortic valves from January 2014 to September 2017 with follow-up through December 2017. | Data for the 31mm CoreValve and the 34mm Evolut R valve and patients with primary aortic insufficiency, with pre-existing surgical or transcatheter valves, and with bicuspid or other nontrileaflet native aortic anatomy. | Not reported. |
| Hellhammer K et al. 2018 [22] | Patients who underwent transfemoral TAVR with the MCV system from September 2015 to January 2018. | Patients with a valve size Evolut R 34mm and Evolut R 23mm. | Not reported. |
| Kalogeras K et al. 2020 [23] | Patients treated with either Evolut R or the Evolut PRO prostheses were included in the study. | Patients requiring the large 34 mm device (annulus perimeter N 81.7 mm) were excluded from the analysis, as the large size prosthesis is only available for the Evolut R platform and not the PRO. All patients with bicuspid valve were also excluded to avoid confounding the results. | Not reported. |
| Kroon HG et al. 2021 [24] | All patients who underwent transfemoral or transsubclavian TAVI with one of the three SEVs for severe aortic stenosis between January 2012 and December 2018 were entered into our prospective database. | Patients with a pacemaker at baseline were excluded ($n$ = 37). | Not reported. |

| | | | |
|---|---|---|---|
| Loewenstein I et al. 2022 [25] | Diagnosis of aortic stenosis based on clinical and echocardiographic criteria of symptomatic patients with an aortic valve area (AVA) of ≤1 $cm^2$ or an indexed AVA of ≤0.6$cm^2$ | Patients with missing valve-type data, treated with valves produced by other manufacturers than Medtronic, patients treated via non-femoral artery vascular access | Choice of senior interventional cardiologist according to valve availability. |
| Modolo R et al. 2020 [26] | Not reported. | Not reported. | Not reported. |
| Rao G et al. 2019 [27] | Severe symptomatic (NYHA ≥2) aortic stenosis (orifice area <1$cm^2$ continuity equation and a mean aortic valve gradient >40 mmHg or a max. velocity >4 m/s at rest on transthoracic echocardiography) in patients deemed high risk for SAVR. | Any previous aortic valve implantation, bicuspid aortic valves and patients requiring subclavian access. | Not reported. |
| Regazzoli D et al. 2019 [28] | Diagnosis of aortic stenosis and small aortic annuli treated with transcatheter implantation of current-generation SEVs (Evolut R, Evolut PRO, Acurate, Portico). A small aortic annulus was defined as annular area <400 $mm^2$ and/or annular perimeter <72 mm on computed tomography. | TAVR for pure aortic regurgitation and a lack of pre-procedural computed tomographic data. | Prosthesis type and size selection, as well as implantation technique and subsequent antithrombotic therapy, were left to discretion of the treating physician at each center. |
| Schmidt S et al. 2022 [29] | Successful TAVR with one of the dedicated valves (S3, ER, Neo), the integrity of pre-procedural CT measurements as well as availability of post-intervention echocardiography. | Patients undergoing valve-in-valve TAVR. | Prosthesis selection was at the discretion of the local heart team at each site. |

**Supplementary Table S4**. Patients' baseline characteristics

| Study [ref] | Intervention | HT (%) | DM (%) | PVD (%) | CKI (%) | COPD (%) | PM/ICD (%) | AF (%) | CAD (%) | MI history (%) | Stroke/CVD history (%) | Heart surgery history (%) | NYHA III/IV (%) | LVEF (%) | Mean aortic valve gradient [mmHg] | Aortic valve area (cm$^2$) | Aortic valve annulus diameter (mm) |
|---|---|---|---|---|---|---|---|---|---|---|---|---|---|---|---|---|---|
| Alvarado T et al. 2021 [19] | EvP | 85 | 21 | 6 | 15 | 9 | 6 | 24 | NR | 3 | 15 | 6 | 48 | 63.0±14.0 | 42.0±13.0 | 0.70±0.16 | 23.0±1.8.0 |
| | EvR | 84 | 34 | 4 | 34 | 16 | 10 | 42 | NR | 8 | 14 | 12 | 68 | 62.0±10,6 | 45.0±18.0 | 0.70±0.18 | 24.0±4.3 |
| Dallan LAP et al. 2021 [20] | EvP | 90 | 32.3 | 25.1 | NR | 44.7 | 2 | NR | NR | 17.6 | 11.9 | NR | 79,7 | NR | NR | NR | NR |
| | EvR | 89.3 | 34.5 | 27.3 | NR | 44 | 3.7 | NR | NR | 21.1 | 13 | NR | 80,4 | NR | NR | NR | NR |
| Forrest JK et al. 2020 [21] | EvP | 92.3 | 37.6 | 26.2 | NR | 41.6 | 16.6 | 35.5 | NR | NR | 12.6 | 18.3 | 75.5 | 56.7±12.9 | 42.4±14.4 | 0.70±0.22 | NR |
| | EvR | 92.3 | 35.8 | 26.8 | NR | 41.7 | 15.2 | 35.5 | NR | NR | 12.8 | 19.5 | 78.1 | 56.6±13.4 | 43.3±15.2 | 0.69±0.21 | NR |
| Hellhammer K et al. 2018 [22] | EvP | 87.8 | 20.2 | 9.5 | NR | 18.9 | 12.1 | 50 | 71.6 | NR | NR | NR | NR | NR | NR | 0.79±0.18 | 73.0±5.4 |
| | EvP | 89.1 | 24.3 | 16.2 | NR | 33.1 | 10.8 | 33.1 | 70.9 | NR | NR | NR | NR | NR | NR | 0.62±0.20 | 73.0±6.2 |
| Kalogeras K et al. 2020 [23] | EvR | NR | 20.7 | NR | 29.3 | NR | 10.7 | 28 | 17.1 | 11.7 | NR | 11.9 | NR | NR | 40.3±19.9 | 0.72±0.48 | NR |
| | EvP | NR | 25.4 | NR | 33.5 | NR | 7.6 | 22.6 | 25.6 | 14.9 | NR | 57 | NR | NR | 46.5±18.8 | 0.69±0.32 | NR |
| Kroon HG et al. 2021 [24] | EvP | NR | 20.7 | NR | 29.3 | NR | 10.7 | 28 | 17.1 | 11.7 | NR | 11.9 | NR | NR | NR | NR | 23.7±1.8 |
| | EvR | NR | 25.4 | NR | 33.5 | NR | 7.6 | 22.6 | 25.6 | 14.9 | NR | 57 | NR | NR | NR | NR | 23.7±1.9 |
| Loewenstein I et al. 2022 [25] | EvP | 90.8 | 37.3 | 8.6 | NR | NR | 13.6 | 27.7 | 36.4 | 15.1 | NR | NR | NR | 58.4±9.7 | NR | 0.67±0.2 | NR |
| | EvR | 86.1 | 37.9 | 10.1 | NR | NR | 13.1 | 28.7 | 44,9 | 15.5 | NR | NR | NR | 56.1±11.0 | NR | 0.69±0.2 | NR |
| Modolo R et al. 2020 [26] | EvP | NR | NR | NR | NR | NR | NR | NR | NR | NR | NR | NR | NR | NR | NR | NR | NR |
| | EvR | NR | NR | NR | NR | NR | NR | NR | NR | NR | NR | NR | NR | NR | NR | NR | NR |

| | | | | | | | | | | | | | | | | |
|---|---|---|---|---|---|---|---|---|---|---|---|---|---|---|---|---|
| Rao G et al. 2019 [27] | EvP | 88.5 | 29.5 | NR | NR | NR | NR | NR | NR | 24.6 | NR | NR | NR | NR | NR | NR | NR |
| | EvR | 94.6 | 37.5 | NR | NR | NR | NR | NR | NR | 17.9 | NR | NR | NR | NR | NR | NR | NR |
| Regazzoli D et al. 2019 [28] | EvP | 81.9 | 24.1 | 19.1 | NR | 3.6 | 14.5 | 25 | 28.6 | 9.6 | 9.5 | NR | 81 | NR | 51.6±1.9 | 0.61±0.02 | 21.4±0.1 |
| | EvR | 85.4 | 26.2 | 18.1 | NR | 13.1 | 9.4 | 20.6 | 38.4 | 11.1 | 8.6 | NR | 74.1 | NR | 50.6±0.8 | 0.65±0.01 | 21.2±0.1 |
| Schmidt S et al. 2022 [29] | EvP | 85.4 | 25 | NR | 54.5 | NR | NR | 31.2 | 56.7 | NR | NR | 7.5 | NR | NR | 48.1±19.5 | NR | 23.4±1.7 |
| | EvR | 89.9 | 30.6 | NR | 62.3 | NR | NR | 43.2 | 62.6 | NR | NR | 17.8 | NR | NR | 34.9±20.7 | NR | 23.8±2.5 |

P, Evolut PRO; R, Evolut R; HT, hypertension; DM, diabetes mellitus; PVD, peripheral vascular disease; CKI, chronic kidney injury; COPD, chronic obstructive pulmonary disease; CVD, cerebrovascular disease; PM/ICD, pacemaker/implantable cardioverter-defibrillator; AF, atrial fibrillation; CAD, coronary artery disease; MI, miocardial infarction; LVEF, left ventricle ejection fraction; NR, not reported.

**Supplementary Table S5.** Procedural characteristics

| Study [ref] | Intervention | Anesthesia (%) | Access site (%) | Valve sizes implanted (%) or mean±SD | Pre-dilatation (%) | Post-dilatation (%) | Contrast volume (ml) | Fluoroscopy time (minutes) | Procedure duration (minutes) |
|---|---|---|---|---|---|---|---|---|---|
| Alvarado T et al. 2021 [19] | EvP | general 45 | femoral 100 | NR | NR | NR | NR | NR | NR |
| | EvR | general 88 | femoral 94 | NR | NR | NR | NR | NR | NR |
| Dallan LAP et al. 2021 [20] | EvP | general 53.5 | femoral 95.4 | 23 - 40.7<br>26 - 37.9<br>29 - 21.4<br>34 - 0 | NR | NR | NR | NR | 110.7±58.5 |
| | EvR | general 59.9 | femoral 95 | 23 - 50.7<br>26 - 33.4<br>29 - 12.6<br>34 - 3.3 | NR | NR | NR | NR | 110.1±57.5 |
| Forrest JK et al. 2020 [21] | EvP | general 59.7 | femoral 94.1<br>subclavian 2.9<br>direct aortic 2.0 | 23 - 3.2<br>26 - 33.6<br>29 - 63.3 | NR | NR | NR | NR | 100.9± 53.9 |
| | EvR | general 55.9 | femoral 93.5<br>subclavian 4.2<br>direct aortic 0.8 | 23 - 4.6<br>26 - 35.4<br>29 - 60 | NR | NR | NR | NR | 109.4± 53.5 |
| Hellhammer K et al. 2018 [22] | EvP | NR | NR | 26 - 51.4<br>29 - 48.6 | 28.3 | 18.9 | 101.7±32.5 | 17.4 ±6.5 | 83.4 ±25.9 |
| | EvR | NR | NR | 26 - 47.3<br>29 - 52.7 | 77.7 | 4.1 | 125.9 ±41.4 | 21.4 ± 8.8 | 105.5±31.2 |
| Kalogeras K et al. 2020 [23] | EvP | general 0 | femoral 100 | NR | 7.4 | 33.8 | NR | NR | NR |
| | EvR | general 26.2 | femoral 94<br>subclavian 5.6 | NR | 9.5 | 21.8 | NR | NR | NR |
| Kroon HG et al. 2021 [24] | EvP | NR | femoral 97 | 23 - 2<br>26 - 33<br>29 - 65 | 12 | 41 | NR | NR | NR |
| | EvR | NR | femoral 89 | 23 - 4<br>26 - 31<br>29 - 49 | 8 | 42 | NR | NR | NR |

| | | | | | | | | | |
|---|---|---|---|---|---|---|---|---|---|
| | | | | 34 - 26 | | | | | |
| Loewenstein I et al. 2022 [25] | EvP | NR | femoral 100 | NR | NR | NR | 124.1±54.5 | 17.5±9.5 | NR |
| | EvR | NR | femoral 100 | NR | NR | NR | 151.9±138.2 | 19.2±11.4 | NR |
| Modolo R et al. 2020 [26] | EvP | NR | NR | NR | NR | NR | NR | NR | NR |
| | EvR | NR | NR | NR | NR | NR | NR | NR | NR |
| Rao G et al. 2019 [27] | EvP | general 98.3 | femoral 100 | 23 - 5<br>26 - 46.7<br>29 - 48.3 | 9.8 | 26.7 | NR | NR | NR |
| | EvR | general 82.1 | femoral 100 | 23 - 3.5<br>26 - 27.3<br>29 - 33.9<br>34 - 30.4 | 5.4 | 25 | NR | NR | NR |
| Regazzoli D et al. 2019 [28] | EvP | NR | femoral 91.7 | 25.8±0.14 | 32.5 | 42.2 | 132.4±6.8 | NR | NR |
| | EvR | NR | femoral 92.4 | 25.8±0.08 | 31.1 | 35.7 | 147.5±4.0 | NR | NR |
| Schmidt S et al. 2022 [29] | EvP | NR | NR | NR | 45.1 | 32.8 | NR | NR | NR |
| | EvR | NR | NR | NR | 30.4 | 26.6 | NR | NR | NR |

EvP, Evolut PRO; EvR, Evolut R; NR, not reported

**Supplementary figures legend:**

**Supplementary Figure S1:** Risk Ratios (RRs) and corresponding 95% Confidence Intervals (CIs) for the comparison of Evolut PRO and Evolut R devices; A) more than 1 valve implanted; B) other TAVI-related complications. Each square represents study point estimate; diamonds reflect the overall effect. IV, inverse variance.

**Supplementary Figure S2**: A) Mean Differences (MDs) and corresponding 95% Confidence Intervals (CIs) for the comparison of Evolut PRO and Evolut R devices in terms of mean transprosthetic gradient; B) Risk Ratios (RRs) and corresponding 95% Confidence Intervals (CIs) for the comparison of Evolut PRO and Evolut R devices in terms of patient-prosthesis mismatch. Each square represents study point estimate; diamonds reflect the overall effect. IV, inverse variance.

**Supplementary Figure S3:** Risk Ratios (RRs) and corresponding 95% Confidence Intervals (CIs) for the comparison of Evolut PRO and Evolut R devices; A) 30-day mortality; B) myocardial infarction; C) cerebrovascular accident. Each square represents study point estimate; diamonds reflect the overall effect. IV, inverse variance.

**Supplementary Figure S4:** Sensitivity analysis for the endpoints moderate-to-severe paravalvular leak (A) and major bleeding (B) after exclusion of single studies one at time and repeating the calculations. Risk difference (RD) analysis for all endpoints including studies reporting “0” events: patient prosthesis mismatch (C); major vascular complications (D); other TAVI related complications (E); 30-day mortality (F); myocardial infarction (G) and stroke (H).

## Supplementary Figure S1:

**A**

| Study or Subgroup | Evolut PRO Events | Evolut PRO Total | Evolut R Events | Evolut R Total | Weight | Risk Ratio IV, Random, 95% CI |
|---|---|---|---|---|---|---|
| Dallan LAP et al. 2021 | 1 | 836 | 20 | 5061 | 6.9% | 0.30 [0.04, 2.25] |
| Forrest JK et al. 2020 | 19 | 1500 | 32 | 1500 | 56.2% | 0.59 [0.34, 1.04] |
| Hellhammer K et al. 2018 | 0 | 74 | 3 | 148 | 3.3% | 0.28 [0.01, 5.42] |
| Kalogeras K et al. 2020 | 0 | 175 | 12 | 498 | 3.6% | 0.11 [0.01, 1.91] |
| Kroon HG et al. 2021 | 4 | 82 | 6 | 160 | 16.8% | 1.30 [0.38, 4.48] |
| Loewenstein I et al. 2022 | 1 | 222 | 10 | 213 | 6.6% | 0.10 [0.01, 0.74] |
| Regazzoli D et al. 2019 | 1 | 84 | 11 | 397 | 6.7% | 0.43 [0.06, 3.28] |
| **Total (95% CI)** | | **2973** | | **7977** | **100.0%** | **0.52 [0.30, 0.89]** |
| Total events | 26 | | 94 | | | |

Heterogeneity: $Tau^2 = 0.05$; $Chi^2 = 6.51$, df = 6 (P = 0.37); $I^2 = 8\%$
Test for overall effect: Z = 2.40 (P = 0.02)

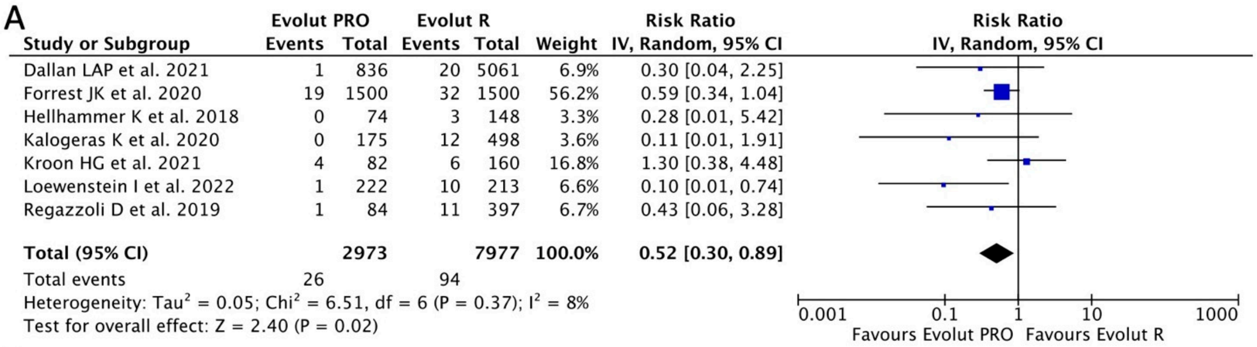


**B**

| Study or Subgroup | Evolut PRO Events | Evolut PRO Total | Evolut R Events | Evolut R Total | Weight | Risk Ratio IV, Random, 95% CI |
|---|---|---|---|---|---|---|
| Alvarado T et al. 2021 | 0 | 33 | 1 | 50 | 9.5% | 0.50 [0.02, 11.92] |
| Dallan LAP et al. 2021 | 1 | 836 | 1 | 5061 | 11.4% | 6.05 [0.38, 96.69] |
| Forrest JK et al. 2020 | 5 | 1500 | 5 | 1500 | 23.1% | 1.00 [0.29, 3.45] |
| Hellhammer K et al. 2018 | 0 | 74 | 3 | 148 | 10.5% | 0.28 [0.01, 5.42] |
| Kalogeras K et al. 2020 | 5 | 175 | 8 | 498 | 24.3% | 1.78 [0.59, 5.36] |
| Loewenstein I et al. 2022 | 2 | 222 | 19 | 213 | 21.1% | 0.10 [0.02, 0.43] |
| Rao G et al. 2019 | 0 | 60 | 0 | 56 | | Not estimable |
| **Total (95% CI)** | | **2900** | | **7526** | **100.0%** | **0.71 [0.22, 2.32]** |
| Total events | 13 | | 37 | | | |

Heterogeneity: $Tau^2 = 1.16$; $Chi^2 = 12.61$, df = 5 (P = 0.03); $I^2 = 60\%$
Test for overall effect: Z = 0.56 (P = 0.57)

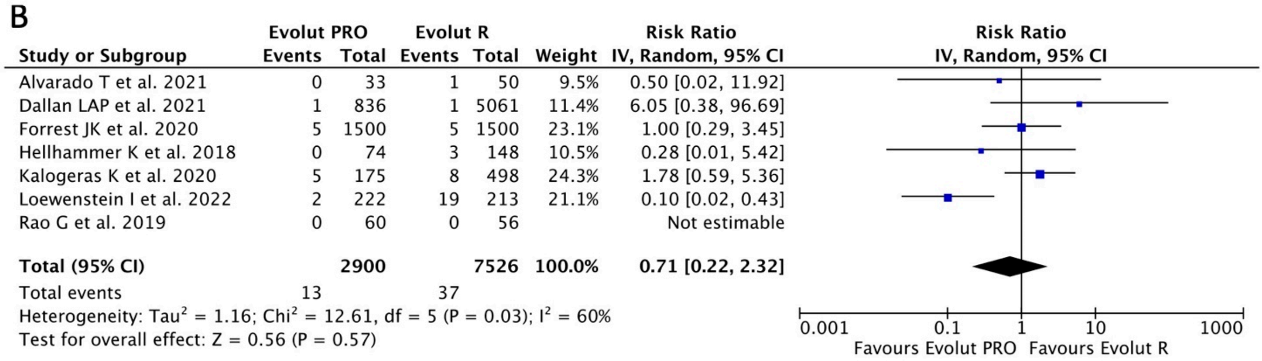


## Supplementary Figure S2

**A**

| Study or Subgroup | EvPRO Mean | EvPRO SD | EvPRO Total | EvR Mean | EvR SD | EvR Total | Weight | Mean Difference IV, Random, 95% CI |
|---|---|---|---|---|---|---|---|---|
| Alvarado T et al. 2021 | 8.9 | 3.7 | 33 | 8.4 | 6.8 | 50 | 6.3% | 0.50 [−1.77, 2.77] |
| Dallan LAP et al. 2021 | 14.9 | 8.4 | 836 | 15.5 | 9.1 | 5061 | 19.0% | −0.60 [−1.22, 0.02] |
| Hellhammer K et al. 2018 | 7.5 | 3.7 | 74 | 7.9 | 3.9 | 148 | 14.6% | −0.40 [−1.45, 0.65] |
| Loewenstein I et al. 2022 | 9.5 | 7.5 | 222 | 8.8 | 5.6 | 213 | 12.8% | 0.70 [−0.54, 1.94] |
| Rao G et al. 2019 | 8.5 | 4.6 | 60 | 8.2 | 4 | 56 | 10.1% | 0.30 [−1.27, 1.87] |
| Regazzoli D et al. 2019 | 6.9 | 3 | 84 | 8.1 | 2 | 397 | 18.5% | −1.20 [−1.87, −0.53] |
| Schmidt S et al. 2022 | 8.4 | 4.5 | 268 | 7.7 | 4.2 | 546 | 18.8% | 0.70 [0.06, 1.34] |
| **Total (95% CI)** | | | **1577** | | | **6471** | **100.0%** | **−0.11 [−0.78, 0.56]** |

Heterogeneity: $Tau^2 = 0.51$; $Chi^2 = 20.50$, df = 6 (P = 0.002); $I^2 = 71\%$
Test for overall effect: Z = 0.33 (P = 0.74)

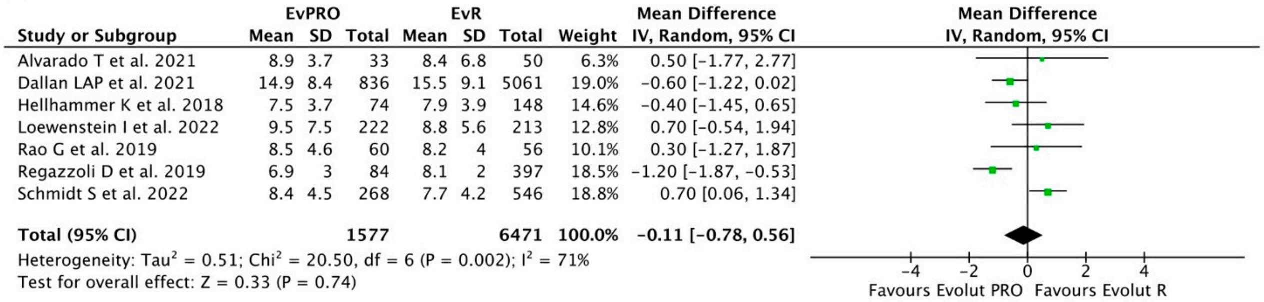


**B**

| Study or Subgroup | Evolut PRO Events | Evolut PRO Total | Evolut R Events | Evolut R Total | Weight | Risk Ratio IV, Random, 95% CI |
|---|---|---|---|---|---|---|
| Dallan LAP et al. 2021 | 356 | 836 | 2035 | 5061 | 96.1% | 1.06 [0.97, 1.15] |
| Rao G et al. 2019 | 0 | 60 | 0 | 56 | | Not estimable |
| Regazzoli D et al. 2019 | 10 | 84 | 67 | 397 | 1.8% | 0.71 [0.38, 1.31] |
| Schmidt S et al. 2022 | 16 | 268 | 32 | 546 | 2.1% | 1.02 [0.57, 1.82] |
| **Total (95% CI)** | | **1248** | | **6060** | **100.0%** | **1.05 [0.97, 1.14]** |
| Total events | 382 | | 2134 | | | |

Heterogeneity: $Tau^2 = 0.00$; $Chi^2 = 1.62$, df = 2 (P = 0.44); $I^2 = 0\%$
Test for overall effect: Z = 1.15 (P = 0.25)

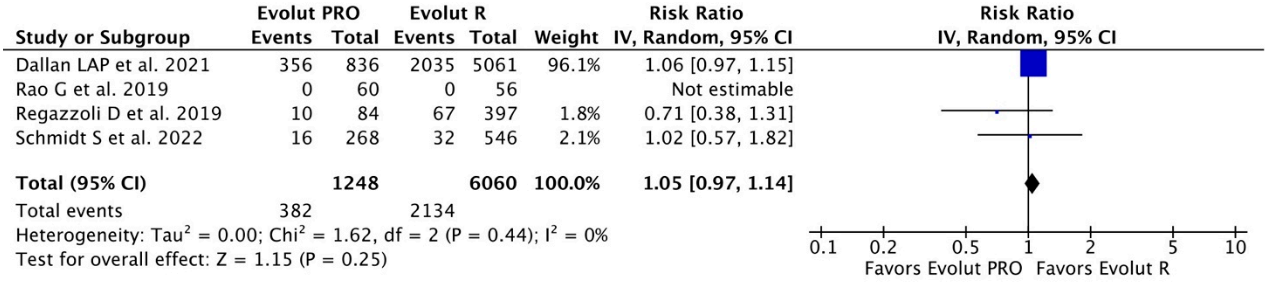

## Supplementary Figure S3

### A

| Study or Subgroup | Evolut PRO Events | Evolut PRO Total | Evolut R Events | Evolut R Total | Weight | Risk Ratio IV, Random, 95% CI |
|---|---|---|---|---|---|---|
| Alvarado T et al. 2021 | 0 | 33 | 3 | 50 | 1.0% | 0.21 [0.01, 4.02] |
| Dallan LAP et al. 2021 | 23 | 836 | 122 | 5061 | 44.4% | 1.14 [0.74, 1.77] |
| Forrest JK et al. 2020 | 37 | 1500 | 44 | 1500 | 46.1% | 0.84 [0.55, 1.29] |
| Hellhammer K et al. 2018 | 1 | 74 | 2 | 148 | 1.5% | 1.00 [0.09, 10.85] |
| Kroon HG et al. 2021 | 1 | 82 | 6 | 160 | 1.9% | 0.33 [0.04, 2.66] |
| Loewenstein I et al. 2022 | 4 | 222 | 5 | 213 | 5.1% | 0.77 [0.21, 2.82] |
| Rao G et al. 2019 | 0 | 60 | 0 | 56 | | Not estimable |
| **Total (95% CI)** | | **2807** | | **7188** | **100.0%** | **0.93 [0.69, 1.25]** |
| Total events | 66 | | 182 | | | |

Heterogeneity: $Tau^2 = 0.00$; $Chi^2 = 3.06$, df = 5 (P = 0.69); $I^2 = 0\%$
Test for overall effect: Z = 0.48 (P = 0.63)

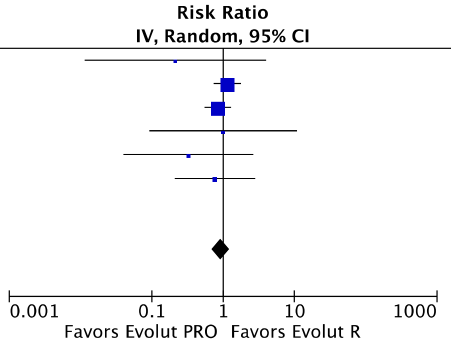


### B

| Study or Subgroup | Evolut PRO Events | Evolut PRO Total | Evolut R Events | Evolut R Total | Weight | Risk Ratio IV, Random, 95% CI |
|---|---|---|---|---|---|---|
| Alvarado T et al. 2021 | 0 | 33 | 0 | 50 | | Not estimable |
| Dallan LAP et al. 2021 | 5 | 836 | 14 | 5061 | 42.0% | 2.16 [0.78, 5.99] |
| Forrest JK et al. 2020 | 6 | 1500 | 2 | 1500 | 28.0% | 3.00 [0.61, 14.84] |
| Hellhammer K et al. 2018 | 0 | 74 | 1 | 148 | 10.5% | 0.66 [0.03, 16.06] |
| Loewenstein I et al. 2022 | 1 | 222 | 5 | 213 | 19.5% | 0.19 [0.02, 1.63] |
| Rao G et al. 2019 | 0 | 60 | 0 | 56 | | Not estimable |
| **Total (95% CI)** | | **2725** | | **7028** | **100.0%** | **1.31 [0.42, 4.05]** |
| Total events | 12 | | 22 | | | |

Heterogeneity: $Tau^2 = 0.53$; $Chi^2 = 5.00$, df = 3 (P = 0.17); $I^2 = 40\%$
Test for overall effect: Z = 0.46 (P = 0.64)

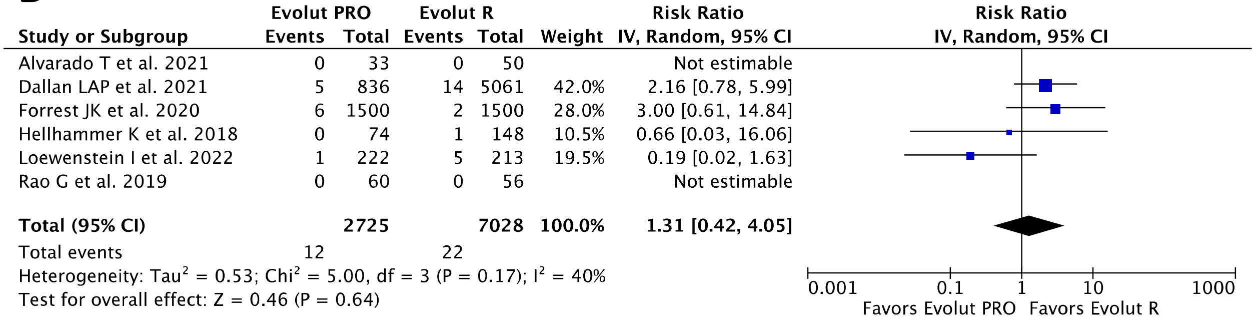


### C

| Study or Subgroup | Evolut PRO Events | Evolut PRO Total | Evolut R Events | Evolut R Total | Weight | Risk Ratio IV, Random, 95% CI |
|---|---|---|---|---|---|---|
| Alvarado T et al. 2021 | 0 | 33 | 0 | 50 | | Not estimable |
| Dallan LAP et al. 2021 | 15 | 836 | 111 | 5061 | 28.7% | 0.82 [0.48, 1.40] |
| Forrest JK et al. 2020 | 48 | 1500 | 58 | 1500 | 58.0% | 0.83 [0.57, 1.20] |
| Hellhammer K et al. 2018 | 1 | 74 | 2 | 148 | 1.4% | 1.00 [0.09, 10.85] |
| Kalogeras K et al. [ATLAS] 2020 | 4 | 175 | 23 | 498 | 7.5% | 0.49 [0.17, 1.41] |
| Kroon HG et al. 2021 | 2 | 82 | 4 | 160 | 2.9% | 0.98 [0.18, 5.22] |
| Rao G et al. 2019 | 2 | 60 | 1 | 56 | 1.5% | 1.87 [0.17, 20.02] |
| **Total (95% CI)** | | **2760** | | **7473** | **100.0%** | **0.81 [0.61, 1.08]** |
| Total events | 72 | | 199 | | | |

Heterogeneity: $Tau^2 = 0.00$; $Chi^2 = 1.42$, df = 5 (P = 0.92); $I^2 = 0\%$
Test for overall effect: Z = 1.45 (P = 0.15)

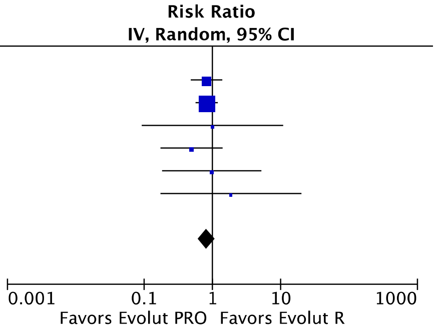

**Supplementary Figure S4:**

# A

| Study name | Point | Lower limit | Upper limit | Z-Value | p-Value |
|---|---|---|---|---|---|
| Alvarado T et al. 2021 | 0.500 | 0.021 | 11.916 | -0.428 | 0.668 |
| Dallan LAP et al. 2021 | 0.463 | 0.220 | 0.973 | -2.031 | 0.042 |
| Forrest JK et al. 2020 | 0.603 | 0.420 | 0.866 | -2.741 | 0.006 |
| Hellhammer K et al. 2018 | 0.599 | 0.419 | 0.858 | -2.793 | 0.005 |
| Kalogeras K et al. [ATLAS] 2020 | 0.588 | 0.424 | 0.816 | -3.179 | 0.001 |
| Kroon HG et al. 2021 | 0.600 | 0.440 | 0.819 | -3.217 | 0.001 |
| Loewenstein I et al. 2022 | 0.606 | 0.449 | 0.818 | -3.275 | 0.001 |
| Modolo R et al. 2020 | 0.605 | 0.455 | 0.805 | -3.451 | 0.001 |
| Rao G et al. 2019 | 0.598 | 0.452 | 0.791 | -3.608 | 0.000 |
| Regazzoli D et al. 2019 | 0.596 | 0.452 | 0.787 | -3.647 | 0.000 |
| Schmidt S et al. 2022 | 0.665 | 0.516 | 0.856 | -3.160 | 0.002 |
| | 0.665 | 0.516 | 0.856 | -3.160 | 0.002 |

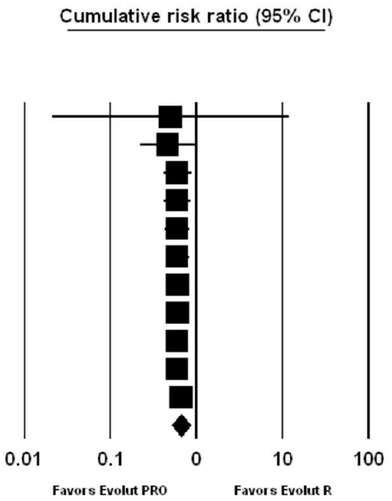


# B

| Study name | Point | Lower limit | Upper limit | Z-Value | p-Value |
|---|---|---|---|---|---|
| Alvarado T et al. 2021 | 0.758 | 0.147 | 3.903 | -0.332 | 0.740 |
| Dallan LAP et al. 2021 | 1.000 | 0.749 | 1.335 | 0.000 | 1.000 |
| Forrest JK et al. 2020 | 0.929 | 0.701 | 1.231 | -0.515 | 0.607 |
| Hellhammer K et al. 2018 | 0.944 | 0.714 | 1.247 | -0.409 | 0.683 |
| Kalogeras K et al. [ATLAS] 2020 | 0.900 | 0.689 | 1.176 | -0.773 | 0.440 |
| Kroon HG et al. 2021 | 0.840 | 0.649 | 1.088 | -1.319 | 0.187 |
| Loewenstein I et al. 2022 | 0.807 | 0.633 | 1.030 | -1.725 | 0.084 |
| Rao G et al. 2019 | 0.816 | 0.641 | 1.038 | -1.657 | 0.097 |
| Regazzoli D et al. 2019 | 0.800 | 0.631 | 1.015 | -1.838 | 0.066 |
| | 0.800 | 0.631 | 1.015 | -1.838 | 0.066 |

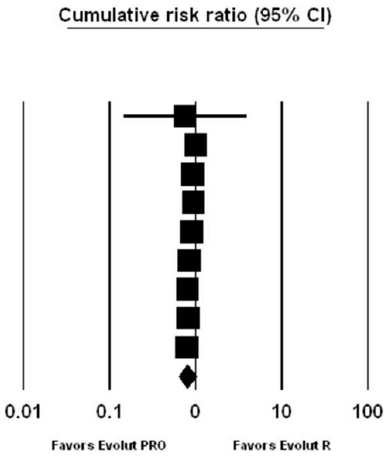

# C

| Study or Subgroup | Evolut PRO Events | Evolut PRO Total | Evolut R Events | Evolut R Total | Weight | Risk Difference IV, Random, 95% CI |
|---|---|---|---|---|---|---|
| Dallan LAP et al. 2021 | 356 | 836 | 2035 | 5061 | 28.6% | 0.02 [−0.01, 0.06] |
| Rao G et al. 2019 | 0 | 60 | 0 | 56 | 34.0% | 0.00 [−0.03, 0.03] |
| Regazzoli D et al. 2019 | 10 | 84 | 67 | 397 | 6.1% | −0.05 [−0.13, 0.03] |
| Schmidt S et al. 2022 | 16 | 268 | 32 | 546 | 31.3% | 0.00 [−0.03, 0.04] |
| **Total (95% CI)** | | **1248** | | **6060** | **100.0%** | **0.00 [−0.02, 0.02]** |
| Total events | 382 | | 2134 | | | |

Heterogeneity: $Tau^2 = 0.00$; $Chi^2 = 3.03$, df = 3 (P = 0.39); $I^2 = 1\%$
Test for overall effect: Z = 0.41 (P = 0.68)

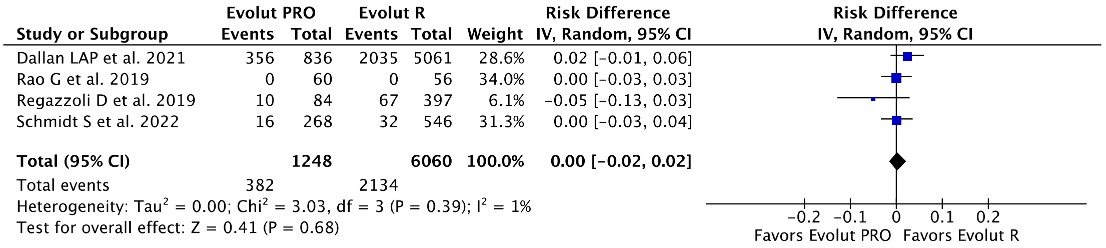


# D

| Study or Subgroup | Evolut PRO Events | Evolut PRO Total | Evolut R Events | Evolut R Total | Weight | Risk Difference IV, Random, 95% CI |
|---|---|---|---|---|---|---|
| Alvarado T et al. 2021 | 2 | 33 | 4 | 50 | 0.6% | −0.02 [−0.13, 0.09] |
| Dallan LAP et al. 2021 | 13 | 836 | 56 | 5061 | 36.3% | 0.00 [−0.00, 0.01] |
| Forrest JK et al. 2020 | 16 | 1500 | 26 | 1500 | 37.7% | −0.01 [−0.02, 0.00] |
| Hellhammer K et al. 2018 | 0 | 74 | 0 | 148 | 14.1% | 0.00 [−0.02, 0.02] |
| Kroon HG et al. 2021 | 4 | 82 | 19 | 160 | 1.6% | −0.07 [−0.14, −0.00] |
| Loewenstein I et al. 2022 | 11 | 222 | 16 | 213 | 3.6% | −0.03 [−0.07, 0.02] |
| Rao G et al. 2019 | 3 | 60 | 3 | 56 | 1.2% | −0.00 [−0.08, 0.08] |
| Regazzoli D et al. 2019 | 2 | 84 | 20 | 397 | 4.8% | −0.03 [−0.07, 0.01] |
| **Total (95% CI)** | | **2891** | | **7585** | **100.0%** | **−0.00 [−0.01, 0.00]** |
| Total events | 51 | | 144 | | | |

Heterogeneity: $Tau^2 = 0.00$; $Chi^2 = 9.65$, df = 7 (P = 0.21); $I^2 = 27\%$
Test for overall effect: Z = 0.97 (P = 0.33)

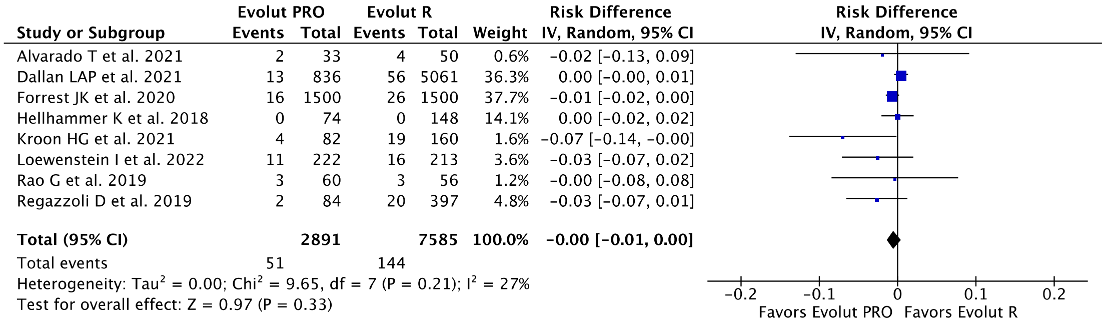


# E

| Study or Subgroup | Evolut PRO Events | Evolut PRO Total | Evolut R Events | Evolut R Total | Weight | Risk Difference IV, Random, 95% CI |
|---|---|---|---|---|---|---|
| Alvarado T et al. 2021 | 0 | 33 | 1 | 50 | 1.4% | −0.02 [−0.08, 0.04] |
| Dallan LAP et al. 2021 | 1 | 836 | 1 | 5061 | 41.0% | 0.00 [−0.00, 0.00] |
| Forrest JK et al. 2020 | 5 | 1500 | 5 | 1500 | 37.8% | 0.00 [−0.00, 0.00] |
| Hellhammer K et al. 2018 | 0 | 74 | 3 | 148 | 5.3% | −0.02 [−0.05, 0.01] |
| Kalogeras K et al. [ATLAS] 2020 | 5 | 175 | 8 | 498 | 6.6% | 0.01 [−0.01, 0.04] |
| Loewenstein I et al. 2022 | 2 | 222 | 19 | 213 | 3.2% | −0.08 [−0.12, −0.04] |
| Rao G et al. 2019 | 0 | 60 | 0 | 56 | 4.6% | 0.00 [−0.03, 0.03] |
| **Total (95% CI)** | | **2900** | | **7526** | **100.0%** | **−0.00 [−0.01, 0.00]** |
| Total events | 13 | | 37 | | | |

Heterogeneity: $Tau^2 = 0.00$; $Chi^2 = 18.63$, df = 6 (P = 0.005); $I^2 = 68\%$
Test for overall effect: Z = 0.71 (P = 0.48)

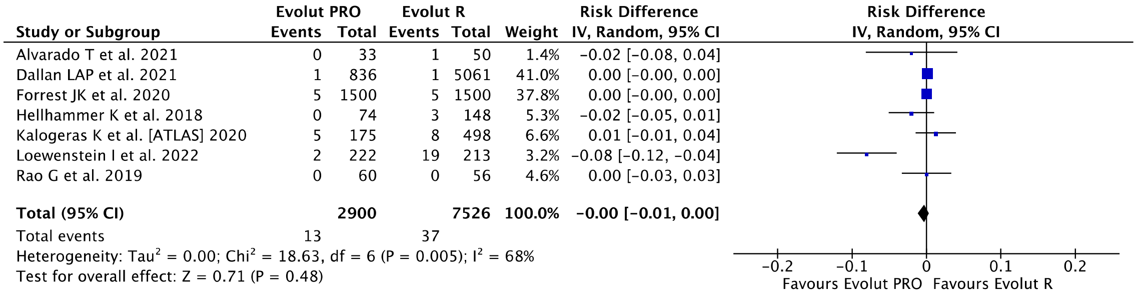


# F

| Study or Subgroup | Evolut PRO Events | Evolut PRO Total | Evolut R Events | Evolut R Total | Weight | Risk Difference IV, Random, 95% CI |
|---|---|---|---|---|---|---|
| Alvarado T et al. 2021 | 0 | 33 | 3 | 50 | 0.8% | −0.06 [−0.14, 0.02] |
| Dallan LAP et al. 2021 | 23 | 836 | 122 | 5061 | 38.1% | 0.00 [−0.01, 0.02] |
| Forrest JK et al. 2020 | 37 | 1500 | 44 | 1500 | 39.8% | −0.00 [−0.02, 0.01] |
| Hellhammer K et al. 2018 | 1 | 74 | 2 | 148 | 5.2% | 0.00 [−0.03, 0.03] |
| Kroon HG et al. 2021 | 1 | 82 | 6 | 160 | 3.7% | −0.03 [−0.06, 0.01] |
| Loewenstein I et al. 2022 | 4 | 222 | 5 | 213 | 7.5% | −0.01 [−0.03, 0.02] |
| Rao G et al. 2019 | 0 | 60 | 0 | 56 | 4.9% | 0.00 [−0.03, 0.03] |
| **Total (95% CI)** | | **2807** | | **7188** | **100.0%** | **−0.00 [−0.01, 0.00]** |
| Total events | 66 | | 182 | | | |

Heterogeneity: $Tau^2 = 0.00$; $Chi^2 = 4.54$, df = 6 (P = 0.60); $I^2 = 0\%$
Test for overall effect: Z = 0.65 (P = 0.52)

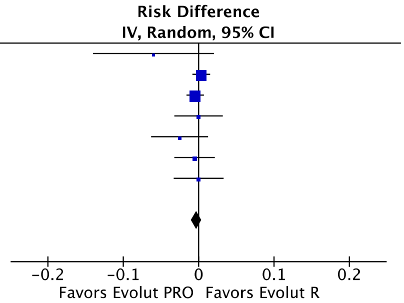


# G

| Study or Subgroup | Evolut PRO Events | Evolut PRO Total | Evolut R Events | Evolut R Total | Weight | Risk Difference IV, Random, 95% CI |
|---|---|---|---|---|---|---|
| Alvarado T et al. 2021 | 0 | 33 | 0 | 50 | 0.4% | 0.00 [−0.05, 0.05] |
| Dallan LAP et al. 2021 | 5 | 836 | 14 | 5061 | 30.2% | 0.00 [−0.00, 0.01] |
| Forrest JK et al. 2020 | 6 | 1500 | 2 | 1500 | 65.3% | 0.00 [−0.00, 0.01] |
| Hellhammer K et al. 2018 | 0 | 74 | 1 | 148 | 1.5% | −0.01 [−0.03, 0.02] |
| Loewenstein I et al. 2022 | 1 | 222 | 5 | 213 | 1.8% | −0.02 [−0.04, 0.00] |
| Rao G et al. 2019 | 0 | 60 | 0 | 56 | 0.8% | 0.00 [−0.03, 0.03] |
| **Total (95% CI)** | | **2725** | | **7028** | **100.0%** | **0.00 [−0.00, 0.01]** |
| Total events | 12 | | 22 | | | |

Heterogeneity: $Tau^2 = 0.00$; $Chi^2 = 4.24$, df = 5 (P = 0.52); $I^2 = 0\%$
Test for overall effect: Z = 1.49 (P = 0.14)

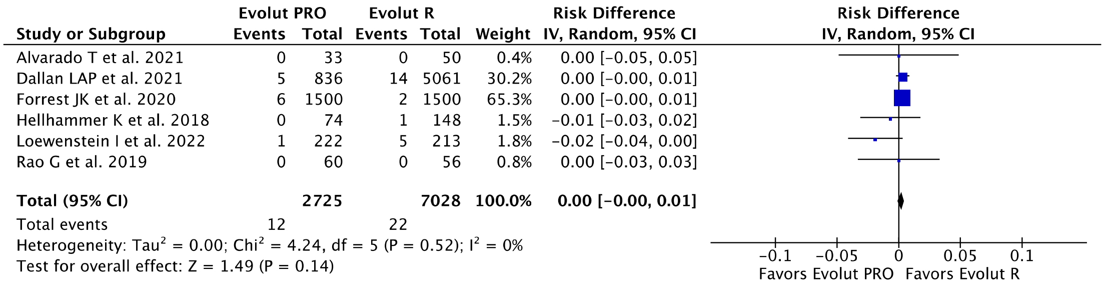

# H

| Study or Subgroup | Evolut PRO Events | Evolut PRO Total | Evolut R Events | Evolut R Total | Weight | Risk Difference IV, Random, 95% CI |
|---|---|---|---|---|---|---|
| Alvarado T et al. 2021 | 0 | 33 | 0 | 50 | 2.2% | 0.00 [−0.05, 0.05] |
| Dallan LAP et al. 2021 | 15 | 836 | 111 | 5061 | 52.7% | −0.00 [−0.01, 0.01] |
| Forrest JK et al. 2020 | 48 | 1500 | 58 | 1500 | 29.4% | −0.01 [−0.02, 0.01] |
| Hellhammer K et al. 2018 | 1 | 74 | 2 | 148 | 4.9% | 0.00 [−0.03, 0.03] |
| Kalogeras K et al. [ATLAS] 2020 | 4 | 175 | 23 | 498 | 6.2% | −0.02 [−0.05, 0.01] |
| Kroon HG et al. 2021 | 2 | 82 | 4 | 160 | 3.0% | −0.00 [−0.04, 0.04] |
| Rao G et al. 2019 | 2 | 60 | 1 | 56 | 1.6% | 0.02 [−0.04, 0.07] |
| **Total (95% CI)** | | **2760** | | **7473** | **100.0%** | **−0.01 [−0.01, 0.00]** |
| Total events | 72 | | 199 | | | |

Heterogeneity: $Tau^2 = 0.00$; $Chi^2 = 2.32$, df = 6 (P = 0.89); $I^2 = 0\%$
Test for overall effect: Z = 1.45 (P = 0.15)

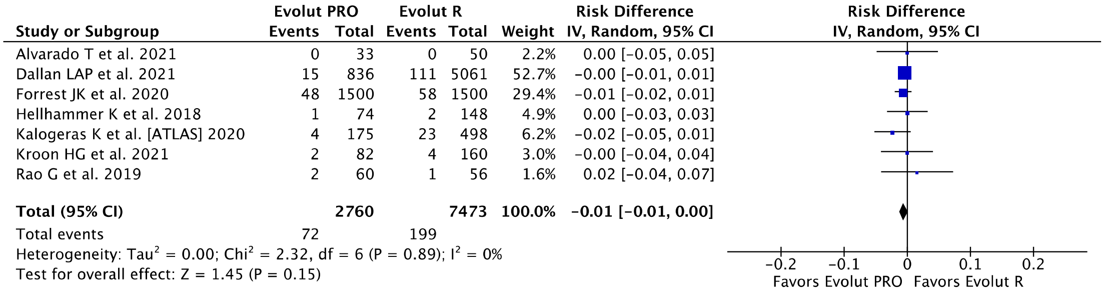

Supplement: Supplementary file 1 [file ijerph-20-03439-s001.zip › ijerph-2169602-supplementary.pdf]
